# Neuron-oligodendrocyte myelination co-culture derived from embryonic rat spinal cord and cerebral cortex

**DOI:** 10.1002/brb3.33

**Published:** 2012-01

**Authors:** Yi Pang, Baoying Zheng, Simpson L Kimberly, Zhengwei Cai, Philip G Rhodes, Rick C S Lin

**Affiliations:** 1 Department of Pediatrics, University of Mississippi Medical CenterJackson, Mississippi 39216; 2 Department of Pathology, University of Mississippi Medical CenterJackson, Mississippi 39216; 3 Department of Neurobiology and Anatomical Sciences, University of Mississippi Medical CenterJackson, Mississippi 39216; 4 Department of Psychiatry and Human Behavior, University of Mississippi Medical CenterJackson, Mississippi 39216

**Keywords:** CNS, myelination, neuron, oligodendrocyte, rat

## Abstract

An in vitro myelination model derived from rat central nervous system (CNS) remains to be established. Here, we describe a simple and reproducible myelination culture method using dissociated neuron-oligodendrocyte (OL) co-cultures from either the embryonic day 16 (E16) rat spinal cord or cerebral cortex. The dissociated cells are plated directly on poly-L-lysine-coated cover slips and maintained in a modified myelination medium that supports both OL and neuron differentiation. The spinal cord derived OL progenitor cells develop quickly into myelin basic protein (MBP)+ mature OLs and start to myelinate axons around 17 days in vitro (DIV17). Myelination reaches its peak around six weeks (DIV40) and the typical nodes of Ranvier are revealed by paranodal proteins Caspr and juxaparanodal protein Kv1.2 immunoreactivity. Electron microscopy (EM) shows typical myelination cytoarchitecture and synaptic organization. In contrast, the cortical-derived co-culture requires triiodothyronine (T3) in the culture medium for myelination. Finally, either hypomyelination and/or demyelination can be induced by exposing proinflammatory cytokines or demyelinating agents to the co-culture, suggesting the feasibility of this modified in vitro myelination model for myelin-deficit investigation.

## Introduction

Myelination is a fundamental biological process in the vertebrate nervous system development. The spiral wrapping by the oligodendrocyte (OL) produced myelin sheath serves not only as a protective layer for axons, but also greatly facilitates the conduction velocity of electrical impulse. Myelination deficits such as hypomyelination, delayed myelination, or demyelination can result in serious motor and cognitive problems seen in many central nervous system (CNS) disorders. The most common myelin-related disorder in premature infants is periventricular leukomalacia (PVL). In this disorder, OLs are damaged and this often leads to hypomyelination or delayed myelination (Leviton and Gilles 1996; Blumenthal 2004; Volpe et al. 2011). As for multiple sclerosis (MS), myelin is attacked and destroyed by autoimmune response, resulting in demyelination and subsequent axonal degeneration (Miller and Mi 2007). As for mechanistic studies of hypomyelination, demyelination, and remyelination, in vitro models are most suitable for such experimentation.

At present, pure OL culture techniques have been well established and extensively used to investigate OL biology (Yang et al. 2005), or to study the mechanisms underlying OL pathology (Pang et al. 2010). As for myelin formation study, one of the most universally used myelination models is the co-culture of purified OLs with dorsal root ganglia cells (Wood et al. 1980; Schnädelbach et al. 2001; Wang et al. 2007). A significant disadvantage of this culture model is that the dorsal root ganglia cells are not CNS neurons. Although several myelination culture models such as the aggregated neuron-OL co-culture (Diemel et al. 2004), brain slice culture (Yang et al. 2011) and explants culture (Chen et al. 2010) from the CNS have been developed, limitations of these models are also noted (Merrill 2009). For instance, the slice cultures are rather thick and they often require a confocal microscope to perform proper examination. The aggregated culture contains multiple layers of cells, making it difficult for the testing reagents and antibodies to access the cultured cells for later quantification. Recently, a dissociated neuron-OL co-culture model from mouse embroynic spinal cord has been described (Thomson et al. 2008). Interestingly, the authors noted also that such culture derived from embryonic rat spinal cord tissue failed to myelinate. Here, we described a novel modified neuron-OL co-culture rat model that can be utilized to investigate the mechanisms of CNS-related myelin deficits.

## Material and Methods

### Chemicals

Dulbecco's modified Eagle Medium (DMEM)/Ham's F12, neural basal medium (NBM), B27 supplement, 7.5% bovine serum albumin (BSA), Hank's Balanced Salt Solution (HBSS), and penicillin/streptomycin were purchased from Invitrogen (Carlsbad, CA, USA). Recombinant rat nerve growth factor (NGF), neurotrophin-3 (NT-3), tumor necrosis factor-α (TNFα), and interleukin-1β (IL-1β), were obtained from R&D system (Minneapolis, MN, USA). Normal horse and fetal bovine serum, insulin, transferrin, sodium selenium, progesterone, putrescine, hydrocortisone, biotin, N-acetyl-L-cysteine, triiodothyronine (T3), L-α-Lysophosphatidylcholine (LPC) were obtained from Sigma-Aldrich (St. Louis, MO, USA). Normal guinea pig serum was from EMD Chemicals (Philadelphia, PA, USA). The sources and specificity of primary antibodies are listed in Table 1. Second antibodies (biotin or fluorescein labeled) were obtained from Jackson ImmunoResearch Lab (West Grove, PA, USA).

**Table 1:** Antibodies used in immunocytochemistry in this study

Antibody name	Source	Dilution	Host	Target(s) of labeling
Myelin basic protein (MBP)	Chemicon	250	Ms	Myelin; mature OL
Neural/Glial antigen 2 (NG2)	Chemicon	400	Rb	Early OL progenitor
OL marker O4	Chemicon	800	Ms	Late OL progenitor
OL transcription factor 2 (Olig2)	Chemicon	500	Rb	OL lineage (progenitor, immature, and mature OL)
Neuronal nuclei (NeuN)	Chemicon	400	Ms	Mature neuron
CD11b	Chemicon	400	Ms	Microglia/macrophage
Glial fibrillary acidic protein (GFAP)	Chemicon	800	Rb	Astrocyte
Contactin-associated protein (Caspr)	Chemicon	200	Rb	Paranodal domain
Potassium channel Kv1.2	Abcam	200	Rb	Juxtaparanodal domain
Phosphorylated neurofilament H (pNF)	Convance	1000	Rb	Mature axon
β-III tubulin (Tuj1)	Sigma	1200	Ms	Neurite (axon and dendrite)

### Myelination co-culture

The dissection of rat E16 spinal cord is similar to that described previously in mice (Thomson et al. 2008). Briefly, spinal cords from six embryos were collected in a petri dish containing 1 mL of 1× HBSS (without Ca^2+^ and Mg^2+^). After carefully removing the meninges, the spinal cord tissue was cut into small pieces using a surgical blade. The minced tissue were then transferred into a 15-mL centrifuge tube with 1 mL Trypsin-EDTA (Sigma #T4299) and incubated for 15 min at 37°C. The enzymatic reaction was stopped by mixing the tissue with 1.5-mL trypsin inhibitor-DNase I solution (0.05% soybean trypsin inhibitor, 0.02% DNase-I, and 0.3% BSA in DMEM), and tissue suspension was centrifuged at 800 *g* for 5 min. The supernatant was replaced with 5-mL plating medium (50% normal horse serum and 20% 1× HBSS with Ca^2+^/Mg^2+^ in DMEM). Tissue was titrated with a 1-mL pipette tip for 10 times. The dissociated cell suspension was then passed through a 40-μm cell strainer. Total number of cells was counted by mixing one part of cell suspension with one part of trypan blue solution. The viable cells typically exceeded 80%. Cells were then seeded on poly-L-lysine-coated cover slips at a density of 0.4 × 10^5^/cm^2^. It is important to note that cell suspension should be spread on the entire top surface of a cover slip sitting in the culture well (300 μl cell suspension for cover slips with 18 mm dimension), but not add into the well directly. After 2-h adhesion, the plating medium was carefully aspirated and myelination medium was slowly added into the wells (700 μl each well for a 12 well plate). The cover slips were firmly pushed down to the bottom of culture wells with a pipette tip. We initially tried either N2 or NBM (with B27 supplement) as the myelination medium, but only limited amount of myelination was observed. However, with the combination of N2 and NBM (1:1) yielded robust myelination in the spinal cord derived culture. For the first week of culture, NGF (50 ng/mL) and NT-3 (10 ng/mL) were included in the medium. The medium was changed every three days by replacing two-third of the medium with fresh medium. The day of the primary culture is defined as day 1 in vitro (DIV1). At DIV10, insulin was excluded from N2 and the ratio of the insulin-free N2 to NBM was adjusted to 4:1 to prevent cell overgrowth. The final concentrations of individual component in N2 medium (DMEM-F12 based, high glucose, Invitrogen) are listed as following: insulin (10 μg/mL), transferrin (50 μg/mL), sodium selenite (5.2 ng/mL), hydrocortisone (18 ng/mL), putrescine (16 μg/mL), progesterone (6.3 ng/mL), biotin (10 ng/mL), N-acetyl-L-cysteine (5 μg/mL), BSA (0.1%), and penicillin–streptomycin (50 units/mL).

The procedures for cortex-derived culture are rather similar to those described from the spinal cord. After removing the meninges and other connective tissue, the entire cerebral cortex from both hemispheres was dissected out and pooled together from six embryos. Typically, total number of dissociated cells from the cortex is much higher (∼10-fold) than from the spinal cord. Under such preparation, T3 was introduced to the myelination medium at DIV10.

### Immunocytochemistry

The cultured cells were rinsed with ice-cold PBS and fixed with 4% paraformaldehyde (PFA) for 15 min at room temperature (RT). Following washing in PBS, cells were permeabilized with 0.5% Triton X-100 for 20 min, and blocked with a solution containing 10% normal goat serum/1% BSA and 0.1% Triton X-100 for 1 h. Cells were then incubated in the primary antibodies diluted in PBS/10% serum overnight. After washing, cells were incubated with biotin- or fluorescein-labeled second antibodies (mouse or rabbit IgG conjugated with Alex 488/555) for 1 h at RT, followed by incubation with avidin fluorescein (Alex 488 or 555) in PBS for 30 min. Cover slips were then washed and air dried, and viewed under a fluorescence microscope (Oly-750 from Olympus, Pittsburgh, PA, USA) with proper filters. For immunostaining of O4, primary antibody was applied before fixation. DAPI (1.5 μg/mL) was used in the mounting medium to counterstain the nuclei. Images were captured with a CCD camera, and superimposed using the Adobe Photoshop (version 7.0) software, if necessary.

### Electron microscopy (EM)

Dissociated cells in the plating medium were seeded into Metrigel Matrix Cell Culture Inserts (BD Biosciences, Bedford, MA, USA), at the same density as that on cover slips. After overnight incubation, the plating medium was replaced with myelination medium (being careful not to disturb cells). Medium change schedule was the same as those in cover slips. At DIV40, cells were fixed with 0.5% glutaraldehyde for 30 min at RT, washed and stored in PBS at 4°C, and then with standard procedures of EM osmication with en bloc, staining of 2% uranyl acetate for 5 min. The tissue was embedded in Durcupan and ultrathin sections were cut and then examined with a LEO Biological transmission electron microscope (Zeiss Corp., Thornwood, NY, USA) equipped with digital camera system for later photograph analysis.

### Treatment with proinflammatory cytokines

At DIV14, the spinal cord derived cells were treated with TNFα (10 ng/mL), IL-1β (10 ng/mL), or the vehicle (1:1000 dilution of PBS in the medium) as the control. The culture medium was exchanged with fresh medium containing TNFα, IL-1β, or the vehicle every 4 days. At DIV40, cells were fixed with 4% PFA and processed for myelin basic protein (MBP)/phosphorylated neurofilament H (pNF) double immunostaining.

### Inducing demyelination

Two commonly used demyelinating reagents were tested on the spinal cord-derived myelination culture at DIV40. For LPC-induced demyelination, cells were treated with LPC (100 μg/mL) or the vehicle (1:1000 dilution of ethanol) serving as the control. For autoimmune-mediated demyelination study, cells were exposed to anti-MOG antibody at 10 μg/mL (Chemicon, Temecula, CA, USA), normal guinea pig serum (source for complement, 12.5 μl/mL) (Diemel et al. 2004), or anti-MOG antibody plus normal guinea pig serum. Untreated sister cultures were used as the control. After 24, 48, and 96 h, cells were fixed and processed for MBP/pNF double immunocytochemistry.

### Quantification of myelination

Two different approaches were conducted to quantify myelination in our cell culture models. First, myelin segments were initially counted manually at DIV26. Ten fields (25× objective) were randomly selected and captured from each cover slip with a fluorescence microscope. The numbers of myelin segments were counted and averaged as one sample. Data were obtained from three separated primary cultures and four cover slips were included in each preparation. Thus, 12 individual samples were obtained for final data analysis. Second, at DIV40, myelin segments were very dense making it almost impossible to individually count the segments. Therefore, an alternative approach was adopted by calculating the ratio of areas occupied by myelinated axons (MBP labeled) to the total area of entire image using ImageJ software (see results). The ratio was defined as myelination index (%).

### Statistics

Statistics were performed using SigmaPlot software (version 11.0). To compare myelination counting between cortex and spinal cord cultures at DIV26, Student's *t*-test was used. Comparisons of myelination among different time points in spinal cord cultures were performed using analysis of variance (ANOVA) followed by post hoc Tukey's analysis. The significant level is set to 0.05.

## Results

### Myelination in the spinal cord derived culture

#### Defining an optimal culture condition

**Figure 1:**
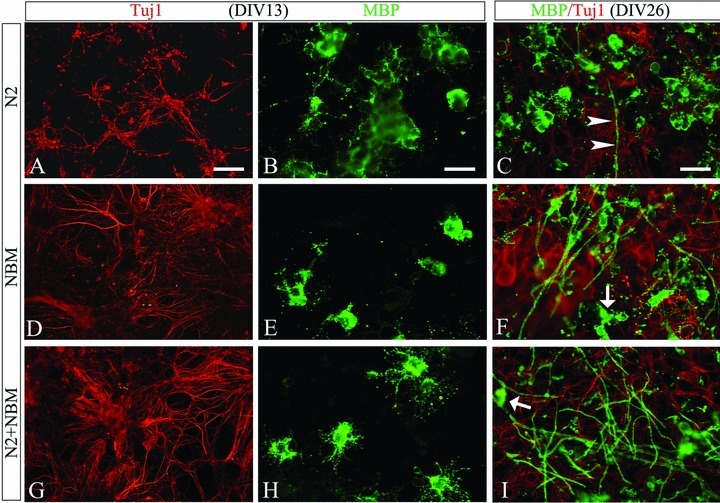
Defining an optimal condition for myelination cultures from E16 rat spinal cord. Cultures maintained in N2 showed poor density of neurite (A), high density of mature OLs (B), but very few myelin segments (arrow heads in C). In contrast, NBM showed a markedly increased neurite density (D), but slight decrease of mature OLs (E), and a moderate increase of myelin segments (F), when compared to N2. A defined medium by mixture of N2 and NBM (1:1) resulted in optimized neurite growth (G), better OL differentiation as shown by highly branched premyelinating OLs (H), and significantly increased number of myelin segments (I). Myelin basic protein (MBP) was downregulated in OL cell bodies at this stage (arrows in F and I). Scale bar: 100 μm (A, D, and G); 25 μm (B, C, E, F, H, and I).

First, we followed in principle the protocol described for the myelination culture derived from embryonic mice spinal cord (Thomson et al. 2008), and N2 was used as the myelination medium. Four weeks later, the culture was double immunostained with MBP (to label myelinated axons, but also labels OL cell bodies and processes) and Tuj1 (to label neurites) antibodies to visualize myelin segments (Fig. 1). In agreement with the previous report, very few myelin segments, if any, were found in cultures derived from the rat spinal cord. The overall density of Tuj1+ neurites was low. However, MBP+ mature OLs were in abundance. Since NBM (with B27 supplement), which is a standard medium for neuronal culture, has been shown to support OL differentiation (Yang et al. 2005), it was then chosen as a substitute for N2. As expected, the density of neurites was indeed significantly improved and the number of MBP+ OLs seemed to be slightly less than in N2 at DIV13. Although myelination was also improved, the numbers of myelin segments remained lower compared to the mice study (Thomson et al. 2008). Interestingly, a combination of N2 and NBM (at a ratio of 1:1) culture medium revealed an extensive number of myelin segments (Fig. 1I) compared to either N2 or NBM alone. This synergetic effect of N2 and NBM on myelin formation appeared primarily due to their improvement on OL development, as most of the premyelinating OLs in N2+ NBM developed longer and finer processes (Fig. 1H) than from either N2 (Fig. 1B) or NBM alone (Fig. 1E).

#### Neuron/glia development and myelin formation

**Figure 2:**
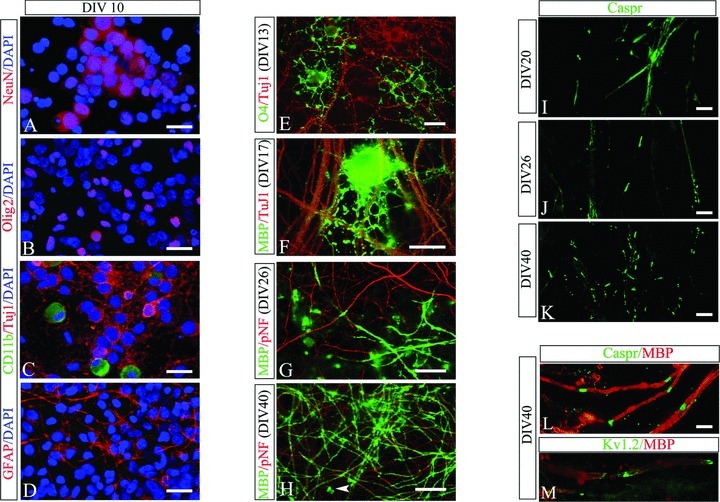
Characterization of myelination culture derived from E16 rat spinal cord. Neurons and glia were identified using their specific markers at DIV10. The major cells types are neurons (A, NeuN+) and OL lineage cells (B, Olig2+), representing more than half of total cells. Microglia/macrophage (C, CD11b+) and astrocytes (D, Glial fibrillary acidic protein [GFAP]+) were mostly found in the periphery of cover slips. Progression of OL maturation and myelination was identified using specific lineage markers. OL progenitor cells (O4+) were highly branched and showed close contact with neurites at early stage (E). At DIV17, premyelinating mature OLs (myelin basic protein [MBP]+) extended their processes and started to wrap around neurites (F). By DIV26, substantial myelin segments, shown as MBP/phosphorylated neurofilament H (pNF) double-labeled lines, were found in the culture (G). Myelination reaches its peak around DIV40 when dense MBP/pNF-labeled axons were shown (H). At this stage, OL cell bodies were only occasionally seen (arrow head in H). The myelination process can also be revealed by the formation of the node of Ranvier. The paranodal protein Caspr initially showed a diffuse pattern of expression on surface of axons at the onset of myelination (I), but became progressively clustered at the nodal region when myelination was active or at its peak (J and K). Higher power view shows the regular spacing of internodes (MBP+) by paranodal domains (Caspr+ in L) or juxataparanodal domains (Kv1.2+ in M). Scale bar: 25 μm (A–H); 10 μm (I–M).

After establishing the optimal culture condition, we next characterized the spinal cord derived myelination co-culture. Since the culture was derived from embryonic rat CNS tissue that contains primarily neural stem cells, our first attempt was to determine the cell phenotypes after neurons and glial cells differentiated. At DIV10, the typical culture contains 38.5% of NeuN+ neurons, 28.3% of Olig2+ OL lineage cells, 10% of Glial fibrillary acidic protein (GFAP)+ astrocytes, and 10% of CD11b+ microglia/macrophage (Fig. 2A–D). In general, neurons were usually found clustered together, and sent their neurites to the areas with a high density of OLs. In contrast, microglia/macrophages and astrocytes were most often found in the peripheral areas of the cover slips, where the density of neurons and OLs was relatively low. Next, we examined OL lineage progression with stage-specific OL markers. Both O4+ late OL progenitor cells and MBP+ mature OLs were found in abundance in the culture after two weeks of culture, while mature OLs became highly branched at the onset of myelination (around DIV17, Fig. 2F). After four weeks, active myelination usually took place as shown by the increased number of myelin segments (co-labeled by MBP/pNF, Fig. 2G). After this time, MBP predominantly labels myelin sheath while its expression was significantly downregulated in OL cell bodies and processes. Myelination in the culture was found at its peak at DIV 40 (Fig. 2H) and then slowed down significantly, but was stable as long as we followed them (∼ three months, data not shown).

Besides the increased number of myelin segments, myelination progression was also evidenced by the expression pattern of Caspr, a paranodal protein that was initially expressed on the surface of unmyelinated axons but became highly clustered at the paranodal domains when mature myelin was formed (Fig. 2I–K). At this late stage of myelination, the typical myelinated internodes were noted as MBP-labeled axons being regularly spaced by multiple nodal domains, that is, Caspr-labeled paranodal domains as well as Kv1.2-labeled juxtaparanodal domains (Fig. 2L and 2M).

#### Ultrastructural characteristics of myelin and synapses

**Figure 3:**
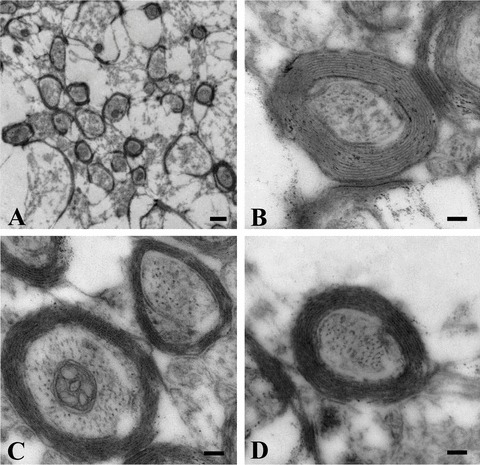
Ultrastructural characteristics of myelination in the spinal cord derived cultures at DIV40. (A) Low power view reveals the distribution of myelinated axons in the culture. Three representative high power photographs show the typical appearance of myelinated axons with extensive myelin sheath wrapped around an axon (B–D). Scale bar: 100 nm (A), 500 nm (B–D).

**Figure 4:**
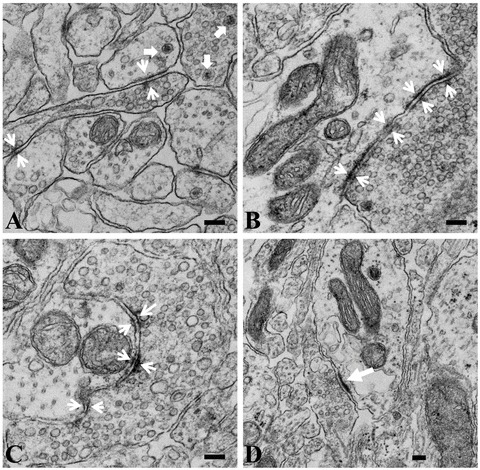
Synaptic specifications in the spinal cord derived co-culture at DIV40. Representative examples of different types of synapses were observed in the culture. Solid arrows in (A) mark the appearance of dense-core vesicles. Apposing small arrows (in A, B, and C) point to synaptic membrane with multiple contact sites. The large solid arrow in (D) points to a typical asymmetric synapse with prominent appearance of postsynaptic density. Scale bar: 100 nm.

The ultrastructural features of both myelin formation and synaptic organization were examined using EM. Extensive and randomly distributed myelinated axons were routinely observed in our samples (Fig. 3A). The integrity of both axons and multiple layer of myelin sheaths were often noted (Fig. 3B–D), suggesting the similarity of our in vitro model with those typically observed in the in vivo models. Furthermore, synaptic organization of both pre- and postsynaptic specifications including synapses containing a variety of different types of vesicles were frequently found (Fig. 4). Examples of dense-core vesicles from several synapses are marked by open arrows in Figure 4A. A variety of typical synapses (Fig. 4B and 4C) including multiple contact sites (Fig. 4B and 4C) asymmetric synapse (Fig. 4D) were also observed.

### Modification of E16 spinal cord culture for cerebral cortex derived culture

**Figure 5:**
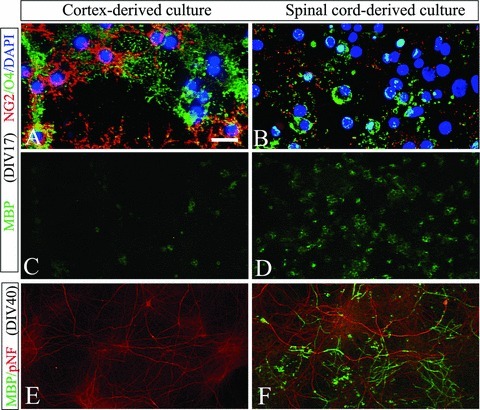
Comparison between the spinal cord and cortex-derived co-cultures. The cortex-derived culture contained more OL progenitor cells (A, NG2+ and O4+) but much fewer mature OLs (C, myelin basic protein [MBP]+) than from the spinal cord (B and D, respectively). Consequently, extensive myelin was formed in the spinal cord derived culture (F), but not the cortex-derived culture (E), although the density of axons appears not significantly different between these two cultures (phosphorylated neurofilament H [pNF] staining in E and F, respectively). Scale bar: 25 μm (A–D); 100 μm (E and F).

Our next goal was to utilize E16 rat cortex for the co-culture model. Surprisingly, simply adopting the spinal cord culture protocol to the cerebral cortex failed to produce discernable myelin formation (Fig. 5E), which was in great contrast to rather abundant myelin formed in the spinal cord derived culture (Fig. 5F). The density of pNF-labeled axons appeared comparable between cultures from these two CNS sources, suggesting that neurons may already well developed in the cortex-derived culture. However, very few MBP+ mature OLs, if any, were found in the cortex-derived culture (Fig. 5C). We then assessed the cell phenotype, and the data showed that total number of OL lineage cells (Olig2+) in the cortex-derived cultures were slightly higher than that from the spinal cord (31.0% vs. 28.3%), indicating OLs in the cortex-derived cultures may fail to mature. This issue was re-examined in cultures at DIV17 (the onset of myelination in the spinal cord derived cultures), and the result revealed that most OL lineages in the cortex-derived cultures remained as either NG2+ early progenitors or O4+ late progenitors (Fig. 5A). In contrast, much fewer OL progenitor cells (Fig. 5B) and more mature OLs (Fig. 5D) were found in the spinal cord-derived culture at this stage.

**Figure 6:**
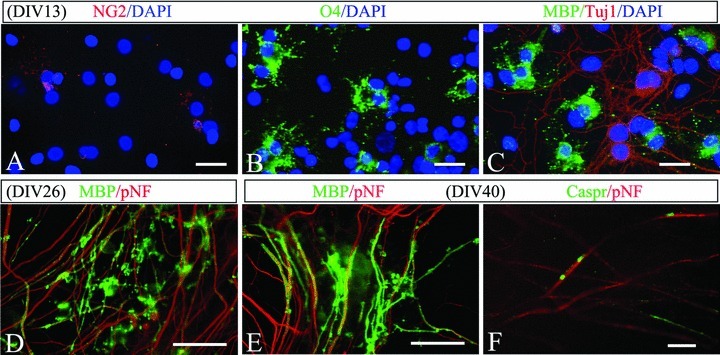
Myelination was significantly increased in the cortex-derived culture by accelerating OL maturation. Three days after T3 was introduced into the medium, the number of NG2+ (A) was reduced while O4+ (B) and myelin basic protein (MBP)+ OLs (C) increased significantly compared to cultures without T3 (see Fig. 5). At DIV 26, active myelination was noted (D), and by DIV40, abundant myelin was formed in the culture (E). The nodes of Ranvier were also evidenced by the clustering of Caspr immunoreactivity (F). Scale bar: 25 μm (A–E); 10 μm (F).

To accelerate OL maturation, we included T3 (60 ng/mL) in the medium starting at DIV10. As expected, OLs matured quickly as shown by a markedly decrease in early OL progenitor cells (NG2+) but an increase of late progenitor cells (O4+) and mature OLs (MBP+) three days after T3 was introduced to the medium (Fig. 6A–C). At DIV26, active myelination was noted (Fig. 6D). At DIV40, myelin segments were in abundance (Fig. 6E) and the nodes of Ranvier were detected (Fig. 6F). Finally, the cell phenotypes in the cortex-derived culture were also determined and compared to that from the spinal cord. In general, the cortex-derived culture contained less neurons (22.3% vs. 38.5%), slightly more OL lineage (31.0% vs. 28.3%), but very few microglia/macrophage (less than 2% vs. 10%) compared to that from the spinal cord.

### Quantification of myelination

**Figure 7:**
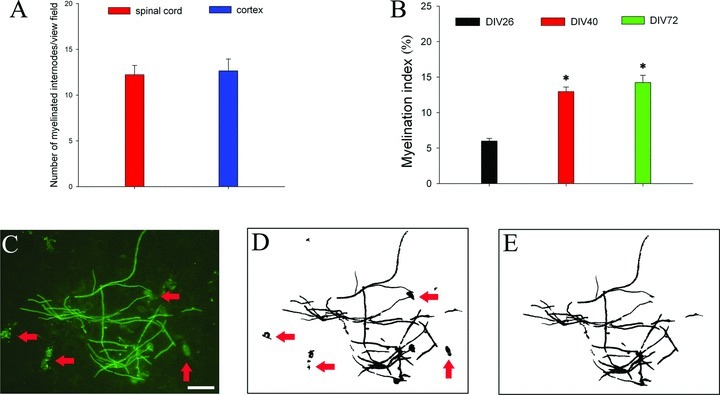
Quantification of myelination. At DIV26, the number of myelin segments was manually counted and further compared between cortex derived cultures and spinal cord-derived cultures, and no significant difference was found between these two cultures (A). At DIV40 or later, when myelin segments were too dense to count, an alternative approach was adopted by calculating the ratio of areas occupied by myelin segments to that of the entire image, and the ratio is defined as myelination index (%). The myelination index was significantly higher at DIV40 compared to DIV26 (*P < 0.05), but no changes were found at later course (B). (C–E) denote how the myelin segments were quantified using ImageJ. (C) is the original image taken for ImageJ analysis (no adjustment), (D) is the image generated by ImageJ outlining the area occupied by myelin basic protein (MBP)+ signals after adjusting the threshold, and (E) is the same image after manually erasing areas occupied by OL cell bodies to leave only myelin segments available for analysis. * *P* < 0.01 vs. DIV26. Scale bar: 25 μm.

An important aspect of the co-culture models is the feasibility to quantify myelin formation at a specified stage. At DIV26, myelin segments can be either manually counted or quantified using ImageJ software (a free software deveoped by NIH, MA). We compared first the myelination between spinal cord and cortex-derived cultures using the manual counting approach. Our data revealed that no significant difference between these two cultures at this relatively early stage of myelination (Fig. 7A). Because of the extensive myelin formation in the culture after DIV26, such manual counting was almost impossible. Thus, at DIV40 or later, myelination was quantified using ImageJ software in order to calculate the percentage of area occupied by MBP-immunostained myelin segments to the total area of the image. An additional advantage of this software program is that it allows us to eliminate the positive signals from OL cell bodies, since they are not part of the myelin structures (illustrated in Fig. 7C, D, and E). With this approach, a more accurate comparison can be made at different DIVs, and our data suggest that myelination appears to reach its threshold around DIV40 (Fig. 7B).

### Reproducibility of the co-culture model

We have performed ten spinal cord and four cortex-derived cell culture (with T3 supplement) preparations. In all these cultures, myelin formation (MBP/pNF double-labeled fibers) has been detected. However, although no difference in myelination at an earlier time-point (i.e., DIV26) was observed between these two tissue-specific cultures, the spinal cord-derived cultures produced more robust myelination at late stages (i.e., DIV40) with better consistency (smaller variation across different preparations). More specifically, the myelin index at DIV40 was 11.5 ± 1.7 (mean ± SD) with a coefficient of variation at 14.8% for the spinal cord derived culture, compared to 8.1 ± 2.1 and 25.9% for the cortex-derived culture (*P* < 0.05).

### Proinflammatory cytokines induce myelin malformation

**Figure 8:**
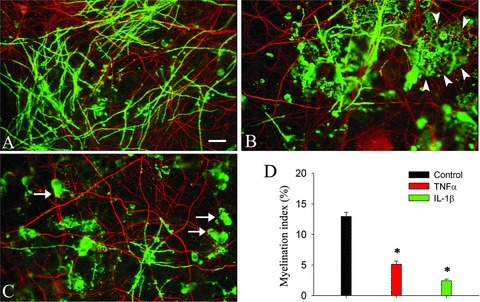
Exposure to TNFα and IL-1β impaired myelin formation in the spinal cord-derived co-culture. The co-cultures were exposed to TNFα and IL-1β (both at 10 ng/ml) or in the medium alone (control) starting from DIV14 until DIV40. Cells were then double immunostained with myelin basic protein (MBP) and phosphorylated neurofilament H (pNF) antibodies. Both TNFα (B) and IL-1β (C) significantly reduced myelin formation when compared to the control (A). Some mature OLs were found to randomly disperse myelin proteins around their cell bodies in TNFα-treated culture (indicated by arrow heads in B). In contrast, an increased number of mature OLs were found in the IL-1 β-treated culture (arrows in C). Data analyzed by ImageJ were shown in (D). Scale bar: 25 μm.

After we established the feasibility and reproducibility of the new co-culture models, our next approach was to validate this new model for mechanistic studies. Our data revealed that myelination in the spinal cord-derived culture was significantly impaired by exposure to both TNFα and IL-1β, with IL-1β-treated cultures appearing more severely affected than TNFα treatment (Fig. 8). In addition to the reduced number of myelin segments, MBP immunoreactive profiles were often found to be dispersed randomly around OL cell bodies in TNFα-treated cultures (arrow heads in Fig. 8B). In contrast, increased numbers of mature MBP+ OLs were often noted in IL-1β-treated cultures (arrows in Fig. 8C). These morphological changes of mature OLs in cytokine-treated cultures were not typically observed in the control (Fig. 8A).

### Demyelination by LPC and autoimmune challenge

**Figure 9:**
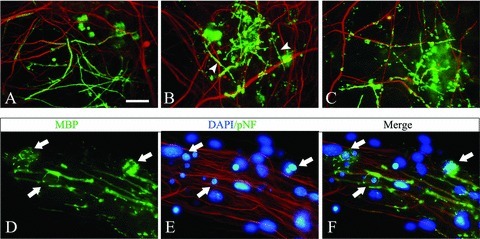
LPC-induced demyelination. The spinal cord derived culture at DIV40 was exposed to LPC (100 μg/mL) and demyelination was examined with myelin basic protein (MBP)/phosphorylated neurofilament H (pNF) double immunostaining. Compared to their intact and smooth appearance in the control (A), most of the myelin sheaths were disintegrated after LPC exposure after three days, although few short myelin segments still appeared to be intact (arrow heads in B). Myelin sheaths were further disintegrated by day 6, and many of them were stripped off the axons (C). An important feature of LPC-induced demyelination is that OLs around demyelination sites were often found degenerative as shown by their condensed and/or fragmented nuclei (arrows in D–F). Scale bar: 50 μm (A–C); 20 μm (D–F).

First, we used LPC to induce demyelination in our culture. Three days after LPC exposure, immunocytochemistry revealed that most of the MBP double-labeled myelin sheaths around axonal fibers lost their integrity (discontinuances and/or irregular), although a few of them remained intact (Fig. 9B). By day 6, myelin sheaths were further disintegrated to become myelin debris (Fig. 9C). An interesting feature of LPC-induced demyelination is that degenerating OLs were often noted by their condensed and fragmented nuclei (Fig. 9D–F).

**Figure 10:**
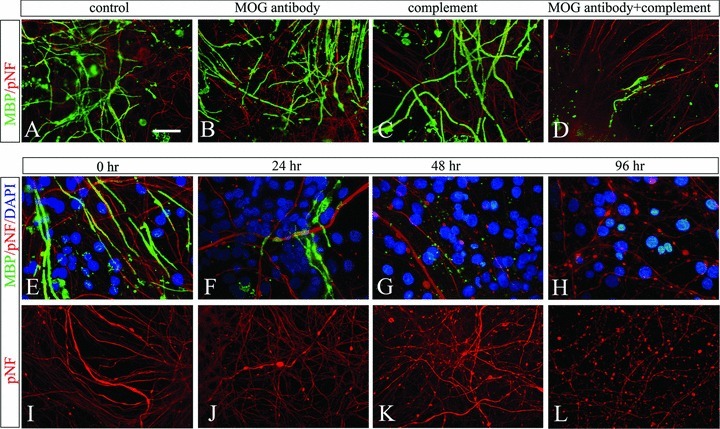
MOG antibody plus complement-induced demyelination. The spinal cord-derived culture at DIV40 was subjected to an autoimmune challenge by exposure to MOG antibody plus complement. Compared to the control (A and E), MOG antibody (B), or complement alone (C), MOG antibody plus complement induced a rapid and severe demyelination. Only a few myelin segments that were rather short and irregular could be found in the MOG antibody plus complement treatment at 24 h (D). At 48 h, only some myelin debris shown as small myelin basic protein (MBP)+ subjects could be detected (G), and they were no long detected at 96 h (H). In addition to demyelination, MOG antibody plus complement also lead to a progressive axonal degeneration (I–L). Scale bar: 50 μm (A–D and I–L); 30 μm (E–H).

We then tested the classic autoimmune-induced demyelination in our cultures. MOG antibody plus complement induced an acute demyelination within 24 h (Fig. 10D). This demyelination was autoimmune specific as it was observed in neither the MOG antibody (Fig. 10B) nor the complement treatment alone (Fig. 10C). Compared to LPC, the autoimmune-induced demyelination was rather rapid and complete. Although very few myelin segments could still be detected in the culture 24 h after the treatment, only occasional myelin debris of MBP+ subjects remained at 48 h, and such subjects were no longer detectable at 96 h (Fig. 10E–H). In addition to myelin damage, few degenerative axons, indicated by their beaded morphology, were also found at 24 h. Such axonal damage became more severe as increasing number of axons with degenerative features were found at 48 h and/or 96 h (Fig. 10I–L). It appears that myelin and axons, but not OLs and /or neurons, were the primary targets of autoimmune attack since cell nuclei remained intact at 96 h (Fig. 10E–H, DAPI counter-staining).

## Discussion

Recently, Thomson et al. (2008) described a dissociated myelination culture model from mice spinal cord but this was unsuccessful using rat CNS tissue. By modifying the culture medium, we have now been able to successfully study the myelin formation in cultures derived from the rat spinal cord and cerebral cortex. Furthermore, we successfully test our new model for myelination deficits commonly used in other models.

Myelination is a fine-tuning biological process that is regulated by a close coordination between OL and neuron/axon. Based on this principle, an idea myelination medium should support a well-balanced development of both OL and neuron/axon, rather than preferentially support one of them. N2 is a traditional cultural medium for OLs and NBM for neurons. When tested individually, they showed poor support for myelin formation. Interestingly, the combination of these two medium produced robust myelination. At present, the precise mechanism for the synergetic effect of N2+ NBM on myelination remains unclear, but it appears that such combination leads to a well-balanced growth and differentiation of neurons and OLs. Furthermore, the OL developmental profile, that is, process extension, rather than the cell number, was noticeably enhanced. In contrast, the neurite density was only moderately improved (Fig. 1). It has been shown previously that process extension is an important step for premyelinating OLs to initially survey the local environment and locate suitable axons (Kirby et al. 2006). The high concentration of insulin in N2 has been shown to activate Akt-mediated survival pathways through the IGF-1 receptor, which is known to promote OL survival and proliferation (D’Ercole et al. 1996; Ebner et al. 2000). In contrast, NBM is known for its antioxidative activity and thus may prevent cell degeneration (Xie et al. 1999). The combination of these two factors may enhance the initial survival and differentiation of neuron stem cells as well as the late specified neurons and OLs. After DIV10, cells survived and myelinated very well in the medium with a lower concentration of insulin (although N2 was insulin free, NBM still contains insulin). The probable explanation is that neurons and glia mutually support each other, since it is well known that both of them can secrete all those factors (Du and Dreyfus 2002; Althaus et al. 2008; Ndubaku and de Bellard 2008). Additionally, those secreted factors have also been suggested to support myelination by affecting OL differentiation (Simons and Trajkovic 2006; Xiao et al. 2009). Taken together, our defined medium is optimal to support neuronal and glial differentiation, resulting in extensive myelination that can be maintained at high levels without any obvious sign of degeneration after long-term culture (∼three months).

An interesting finding in this study is that the mechanism of myelination appears to differ in cultures derived from the spinal cord versus cerebral cortex. The failure of myelination in the cortex-derived culture may be due to arrest in OL differentiation, since accelerating OL maturation by T3 resulted in a high level of myelination. The striking difference of OL development in these two CNS-derived cultures may be due to the intrinsic difference in OL differentiating potential, and/or differences in extrinsic factors produced by neurons and glia. Recent studies, for example, have suggested that OL differentiation is regulated by both an intrinsic clock that turns on in OL progenitor cells after certain divisions, and also by extrinsic cues provided by neighboring neurons and glia (Emery 2010). Furthermore, OLs in the spinal cord are mostly derived from the subventricular zone (SVZ) and migrate through the spinal cord and differentiate into mature OLs (Sun et al. 1998), while OLs from forebrain exhibit three different waves of OL progenitors that are generated from different origins (Kessaris et al. 2006; Richardson et al. 2006). In addition, the behaviors of competition for growth factors are also different in OL progenitors derived from the spinal cord versus the cerebral cortex (Bradl and Lassmann 2010). Therefore, it is quite possible that the intrinsic potential of differentiation in the spinal cord derived OLs is much greater than that from the cortex. Interestingly, our data also suggested that the cell phenotypes may also be different between these two CNS-derived cultures, and they may also contribute to the disparity noted in OL maturation between these two cultures. Lastly, recent studies have suggested that neuronal/axonal factors (i.e., adhesion molecules expressed on axonal surface, electrical activity, size, etc.) may play important roles in controlling myelination (Piaton et al. 2010). The difference between neuronal phenotype, that is, predominantly sensory and motor neurons in the spinal cord versus a diversity of neurons in the cerebral cortex may also account for the difference in myelination potential. Nevertheless, when OL progenitors were forced to mature by T3, successful myelination occurred in the cortex-derived culture, suggesting that the lack of OL maturation may be the major cause of myelination failure in the cortex-derived culture.

Nodes of Ranvier are important structures of myelinated axons that ensure the propagation of rapid, saltatory nerve conduction. The nodes are comprised of several subdomains including the node, paranode, and juxtaparanode regions that can be identified with specific markers (Southwood et al. 2004; Simons and Trajkovic 2006). Using paranodal marker Caspr and juxtaparanodal marker Kv1.2, our data revealed that the typical nodes of Ranvier were successfully constructed. In addition to myelination, abundant synapses with a variety of specification were also found ultrastructurally, suggesting that our culture system recapitulates the developmental features similar to the in vivo environment.

Another feature of our myelination culture system is that quantification of myelination can be conducted using both the manual counting and ImageJ approaches. The direct quantification of myelin segments (although we only measured the number, the length can also be determined) can provide additional information other than quantifying the amount of myelin proteins. For instance, early studies using in vitro myelination models, for example, the aggregate culture, measured the amount of myelin proteins (e.g., MBP) as an index for myelination (Diemel et al. 2004). This approach could be inaccurate and/or even misleading, since our cytokine-treated culture revealed that MBP is upregulated in cell bodies (IL-1β) or deposit randomly in the surrounding vicinity (TNFα) (Fig. 8). Thus, the changes in total MBP (located in both mature OL cell bodies/processes and myelin sheaths) may not accurately represent myelin formation.

Our data suggested that the myelination models from both tissue sources are rather reproducible; however, the variation across different cell culture preparations appeared to be higher in the cortex-derived cultures. This slight difference may be partially attributed to the method that we used for myelin quantification. In the spinal cord derived cultures, most MBP+ subjects were myelinated fibers but not OL cell bodies and processes, therefore, the quantifications were rather straightforward. In contrast, since both the number and intensity of OL cell bodies/processes immunostained with MBP increased in the cortex-derived cultures and they need to be manually deleted before the areas measurement by ImageJ software, it is not surprising that variations are more noticeable.

Our next goal was to validate the myelination culture as a potential in vitro model to study hypomyelination and demyelination. First, we demonstrated that TNFα and IL-1β, two of the proinflammatory cytokines that have been implicated in mediating hypomyelination in PVL (Pang et al. 2003; Huleihel et al. 2004), significantly impaired myelination in the spinal cord derived culture. Both TNFα and IL-1β were used at relatively low concentration (10 ng/ml) but with prolonged exposure to mimic the in vivo scenario. Interestingly, the markedly impaired myelination was also associated with changes in OL behavior, that is, random disperse of MBP by OLs in TNFα-treated culture and increased reactivity of MBP in OL bodies in IL1β-treated culture, rather than directly damage OLs, suggesting that proinflammatory cytokines may interfere with myelination by changing OL functions. It is now recognized that myelination is a rather complicated process that is regulated at many levels, it not only depends on the quality and quantity of mature OLs that can be affected by any alterations in OL development (i.e., OL specification, balance between proliferation and apoptosis, migration, and differentiation; Piaton et al. 2010), but also signals derived from neurons/axons (i.e., adhesive molecules, Notch pathway, LINGO-1) (Fancy et al. 2011; Kotter et al. 2011). For these aspects, our cell culture model will be very useful in investigating the role of both OLs and axons that may contribute to myelination deficit following exposure to certain putative insults (cytokines, oxidative damage, hypoxia-ischemia, etc.) relevant to PVL.

Finally, demyelination was successfully reproduced in the myelination co-culture by exposure to LPC and MOG antibody plus complement, two widely used demyelinating insults. In the present study, LPC-induced demyelination was observed at least three days after the exposure and obviously involved in OL degeneration (Fig. 9), this finding is consistent with the fact that LPC causes demyelination by primarily damaging OL (Fressinaud 2005). In contrast, MOG antibody plus complement showed a rapid (within 24 h), complete demyelination, as the myelin sheath can be directly damaged by this autoimmune reaction (Keirstead and Blakemore 1997).

In summary, we describe a simple yet reproducible protocol for in vitro myelination culture derived from rat CNS tissue. Our model may be utilized for mechanistic studies such as hypomyelination and/or demyelination.
